# Salvage Proton Therapy Re-Irradiation in Recurrent Head and Neck Cancer: Outcomes and Adverse Events by Re-Irradiated Target Site

**DOI:** 10.3390/cancers18142207

**Published:** 2026-07-09

**Authors:** Enrique Amaya, Jacobo Palma, Roser Fayós-Solà, Rosa Meiriño, Mauricio Cambeiro, Ana Navarrete, Pablo Cabello-García, Alberto Viñals, Diego Pedrero, Felipe A. Calvo, Javier Aristu, Javier Serrano

**Affiliations:** 1 Department of Radiation Oncology, Clinica Universidad de Navarra, 28027 Madrid, Spain; jpalmadelga@unav.es (J.P.); rmeirino@unav.es (R.M.); mcambeiro@unav.es (M.C.); anavarretet@unav.es (A.N.); fcalvom@unav.es (F.A.C.); jjaristu@unav.es (J.A.); fserranoa@unav.es (J.S.); 2 Department of Medical Physics, Clinica Universidad de Navarra, 28027 Madrid, Spain; rfayossola@unav.es (R.F.-S.); jpcabello@unav.es (P.C.-G.); avinalsmu@unav.es (A.V.); dpedrerodea@unav.es (D.P.)

**Keywords:** re-irradiation, proton therapy, IMPT, head and neck cancer, recurrent, adverse event, overall survival, locoregional control

## Abstract

**Background/Objectives:** Photon re-irradiation in previously treated head and neck cancer (HNC) is constrained by cumulative normal-tissue toxicity. Pencil-beam scanning intensity-modulated proton therapy (PBS-IMPT) allows for a sharper dose conformation than conventional photon techniques, which may widen the therapeutic window in previously irradiated tissue. **Methods:** Sixty-five patients with recurrent or second primary HNC were enrolled in a prospective single-institution registry and re-irradiated with PBS-IMPT between January 2020 and December 2025. Forty-five were treated with radical intent (69.2%), and 20 were treated postoperatively (30.8%), with a median prescribed dose of 66 Gy (RBE) in 30 fractions. Primary endpoints were overall survival (OS) and progression-free survival (PFS). Locoregional control (LRC), adverse events and the prognostic impact of anatomical extent were secondary endpoints. **Results:** The median follow-up was 9.3 months. The median OS was 17.2 months (95% CI 13.9–NR), with a 12-month rate of 67.1%. The median PFS was 10.1 months and the median LRC was 28.1 months. Central/skull-base involvement was associated with a non-significant trend toward worse OS (14.3 vs. 35.2 months; *p* = 0.062) and with significantly worse PFS (9.1 vs. 14.7 months; *p* = 0.037) than peripheral disease, but LRC did not differ between the groups (*p* = 0.240). Grade ≥ 3 oral mucositis occurred in 7.7%, with no grade 4–5 acute events. Within a median follow-up of 9.3 months, late osteoradionecrosis affected 6.2%, but late toxicity data remain preliminary given the short follow-up. Ninety-two percent of patients completed treatment. **Conclusions:** PBS-IMPT re-irradiation provided adequate survival with low acute toxicity. T3–T4 stage at re-irradiation was the only variable retaining significance for PFS on multivariable analysis (HR 4.32, 95% CI 1.24–15.13; *p* = 0.022). The poor survival observed for central/skull-base disease on Kaplan–Meier curves disappeared after T-stage adjustment, indicating the higher concentration of advanced disease in that compartment. Multicentre prospective data and longer follow-up are needed.

## Simple Summary

Head and neck cancer that recurs inside a prior radiation field is difficult to manage, and treatment options are limited. Photon re-irradiation can control disease but is associated with notable toxicity. Pencil-beam scanning intensity-modulated proton therapy (PBS-IMPT) concentrates the dose at the target and spares what lies beyond, which is important in already-irradiated tissue. We treated 65 patients over 5 years. The median survival was 17.2 months; fewer than 8% of patients developed severe mucositis. Central/skull-base recurrences were associated with shorter survival than peripheral recurrence, although local control did not differ significantly between groups. The multivariable model identified T-stage as the main explanation: these tumours simply concentrated more advanced disease. These results support a selective role for PBS-IMPT in carefully selected patients with recurrent head and neck cancer.

## 1. Introduction

Locoregional recurrence develops in 15–50% of patients treated for head and neck cancer (HNC), with the precise figure varying with the primary site, stage at presentation and initial treatment [1,2]. When relapse occurs within the prior radiation field, the available options are limited. Surgery is the preferred salvage option when the disease is resectable and the patient is fit; however, a large proportion of patients cannot be offered this path. This includes those whose tumours have grown beyond resectable limits, those whose performance status makes a major procedure unsafe, and those for whom a prior resection has already been attempted without success. Systemic therapy alone offers only modest palliation in this population [2,3]. Re-irradiation is the only modality that offers a realistic possibility of cure or prolonged disease control in unresectable cases of recurrence. The main issue is the potential toxicity in the tissue and the organs at risk (OARs). Previously irradiated brainstem, spinal cord, temporal lobes, carotid arteries and mandible leave little margin before cumulative doses become dangerous. Photon re-irradiation series, including both Intensity-Modulated Radiotherapy (IMRT) and Stereotactic Body Radiotherapy (SBRT), report grade 3–5 toxicity in 20–50% of patients, with fatal complications such as carotid blowout occurring in a small proportion [4,5,6,7,8]. The MIRI collaborative (*n* = 412), probably the most robust photon re-irradiation dataset available, reported 2-year overall survival (OS) of 17–62% across risk groups [9]. This shows that re-irradiation is a complex and delicate process that requires careful consideration. Proton therapy (PT) has an apparent theoretical advantage in this context. The Bragg peak allows the dose to stop at the target rather than continuing through downstream normal tissue [10]. In re-irradiation, where every Gray administered to the OARs is critical, this is not a trivial difference [11]. Several institutional series confirm that proton re-irradiation achieves outcomes comparable to or better than those of photon modalities [12,13,14]. Lee et al. reported a 1-year OS of 66.6% and locoregional control (LRC) of 71.8% in 242 patients at Memorial Sloan Kettering [15]. Romesser et al. and Phan et al. published multi-institutional findings with similar results [12,13]. Systematic analyses consistently show a late toxicity advantage for protons over photons in re-irradiation [11,16,17,18]. Most proton re-irradiation series have come from North American high-volume centres and use an earlier generation of PT technology [17]. Pencil-beam scanning (PBS)-based intensity-modulated proton therapy (IMPT), which provides finer dose modulation, is sparsely represented in the re-irradiation literature [14,17], and European experience is particularly scarce [19]. Whether results from large North American centres translate to the European context, with its different referral patterns and patient profiles, is a valid question. We report the outcomes of 65 consecutive patients treated with PBS-IMPT re-irradiation at a single European proton centre.

## 2. Materials and Methods

### 2.1. Study Design and Patient Population

Between January 2020 and December 2025, all patients with recurrent or second primary HNC who underwent re-irradiation with PT at our centre were prospectively enrolled in an institutional registry. The registry collects clinical, dosimetric and follow-up data as part of routine clinical care. This study applied retrospective analytical methods to this prospectively maintained cohort. Eligibility was limited to patients over 18 years old with a documented prior course of head and neck radiotherapy. Prior irradiation was not delivered under a uniform protocol, as patients were referred from multiple centres; prior treatment characteristics were therefore heterogeneous. Most patients (*n* = 55, 84.6%) had received a single prior course, nine had been irradiated twice and one three times. The prior technique was VMAT/IMRT in 93.8% of cases, BT in three cases and PT in one case; the mean cumulative prior dose was 66 Gy (range 24–80.5), delivered predominantly with conventional fractionation (median 2.0 Gy/fraction) apart from a few hypofractionated or stereotactic schedules. The median interval between the last RT course and PT re-irradiation was 34 months (range 11–300). All included patients provided written informed consent for the collection and storage of their clinical and dosimetric data, and for their use in retrospective research analyses and scientific publications. All data were anonymised before analysis.

The disease was classified according to its location and histology as recurrent disease or a second primary tumour. Recurrent disease was defined as a tumour in the same or adjacent area that was treated before with RT, and a biopsy was performed with a histological result consistent with that of the original primary tumour. A second primary tumour was defined as a tumour with different histology or anatomical subsite, or occurring more than 5 years after the initial diagnosis, according to the MIRI Collaborative criteria [9].

The anatomical site of recurrence in HNC can significantly affect the feasibility of re-irradiation, the dose that can be delivered to the clinical target volume (CTV), and the cumulative dose to surrounding OARs. To account for these differences in dosimetric and patient outcomes, and to make subgroup analysis more meaningful, patients were grouped based on the anatomical extension of the re-irradiation area. This helped to determine how well the CTV was covered, the dose the surrounding OARs received, and the overall outcomes for patients with tumours in different locations. For the anatomical classification, patients were divided into three groups: central/skull-base, unilateral and bilateral extension. We collapsed these into two main groups for comparative analysis: central/skull base (including all cases with a skull-base component) and peripheral (essentially unilateral or bilateral without central/skull-base involvement). The central/skull-base group comprised nasopharyngeal and paranasal sinus tumours, together with any lesion reaching the orbit, masticator space, cavernous sinus, anterior cranial fossa, clivus or regions adjacent to the brainstem. The peripheral group involved the oral cavity, oropharynx, larynx, parotid gland or cervical lymph nodes.

### 2.2. Treatment Planning and Dose

All patients were treated with PBS-IMPT at a Hitachi Probeat-CR facility (Hitachi Ltd., Tokyo, Japan) with plans generated in the RayStation version 12A (Raysearch Laboratories AB, Stockholm, Sweden) treatment-planning system using a Monte Carlo dose engine. Immobilisation relied on individualised thermoplastic masks, and planning CT was acquired with a slice thickness of 1–1.5 mm. MRI and PET-CT in the treatment position were obtained in every case to support target and OAR delineation.

Proton beams are sensitive to small physical and geometric variations along the beam path, including tissue heterogeneities, day-to-day positioning and anatomical changes during the course of treatment. Beam angle selection was used to minimise the effects of anatomical changes during treatment, while robust optimisation at the planning stage was applied to account for setup and range uncertainties. Robust optimisation was performed directly on the CTV, incorporating a setup uncertainty of ±3 mm in isocentre shifts and a proton range uncertainty of ±3.5%; the range value was calibrated for our planning CT scanner [20].

**Figure 1:**
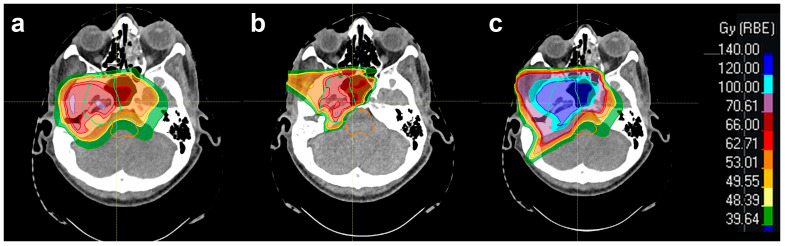
Axial dose distribution in a representative re-irradiation case. (**a**) Previously delivered photon dose; (**b**) proton re-irradiation plan (PBS-IMPT); and (**c**) cumulative EQD2 dose distribution, illustrating the dose contribution from both treatment courses to the organs at risk.

To account for previous irradiation exposure, hybrid deformable registration based on the anatomical constrained deformation algorithm (ANACONDA) between the previous planning CT and the current simulation CT was performed, allowing dose accumulation and transfer of the previously delivered dose distribution onto the current anatomy. The combined dose was expressed as the equivalent dose in 2-Gy fractions (EQD2, α/β = 2 Gy for late-responding normal tissues) (Figure 1). OAR constraints were individualised based on the estimated cumulative dose from prior irradiation, with particular attention to the brainstem, spinal cord, optic pathway, temporal lobes, mandible and carotid arteries, considering current evidence on cumulative dose-toxicity relationships in the re-irradiation setting [21,22].

**Figure 2:**
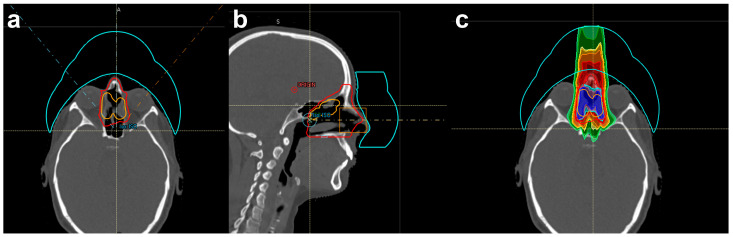
Beam configuration and dose distribution with patient-specific bolus for cancer patients treated with proton therapy. (**a**,**b**) Axial and sagittal CT views of a two-field arrangement for central tumour localisation, using a custom bolus. (**c**) Corresponding dose distribution.

For the planning workflow, we maintained the same anatomical split used for the comparative outcome analysis—central/skull base versus peripheral—so that the planning approach and the clinical results could be directly read against each other. For the first group, we used between two and three beams with a patient-specific bolus that was attached to the mask covering the entire beam entrance area (Figure 2). For the second group, as we could not attach the bolus to the mask in those regions, we used between two and three beams, with the option of incorporating a range shifter (RS) in one or more of them if needed. Both the bolus and range shifter were used to ensure adequate dose coverage of the most superficial portions of the CTV.

Prescribed doses ranged from 45 to 72.6 Gy (RBE) with a median of 66 Gy (RBE). Fractionation was selected based on clinical decisions. Conventional fractionation was used in most patients. The most common schedule, 2.2 Gy (RBE) per fraction in 30 fractions, accounted for a third of the cohort (33.8%), and another 21.5% received 2.0 Gy (RBE) per fraction over the same number of fractions. Fraction sizes in the remaining patients ranged from 1.8 to 2.5 Gy (RBE) across 25–35 fractions, and one patient was treated with a hypofractionated schedule of 5 Gy (RBE) in 10 fractions. CTV delineation was based on contouring gross tumour volume (GTV) with anatomically adjusted margins. High-risk CTV includes the gross tumour (or, in postoperative cases, the surgical bed with microscopically involved or close margins) and receives a dose greater than 60 Gy (RBE). Intermediate-risk CTV covers areas of microscopic disease extension, and the prescribed dose is up to 50–60 Gy (RBE).

### 2.3. Systemic Therapy

In some cases, neoadjuvant chemotherapy (ChT) was administered according to multidisciplinary tumour board recommendations of the referring centre, with the overall goal of achieving tumour downstaging or improving local control before re-irradiation. The regimens were heterogeneous, reflecting the variability of protocols among referring institutions. Cisplatin-based regimens were predominant, followed by carboplatin-based schedules, which were generally reserved for patients with contraindications or intolerance to cisplatin. Concurrent ChT was administered considering the recommendations of the referring centre and our oncology committee decisions. No patients received both neoadjuvant and concurrent treatment. Because regimens were prescribed by the referring multidisciplinary tumour boards rather than under a single institutional protocol, they were heterogeneous; cumulative cytotoxic doses and formal treatment compliance were therefore not uniformly recorded. The agents used and the number of cycles administered are detailed in Section 3.1. Systemic therapy was predominantly ChT; immune checkpoint inhibition (one patient with concurrent atezolizumab) and targeted agents were exceptional. Neither prior nor concurrent systemic therapy altered the re-irradiation prescription, fractionation, robust optimisation strategy or OAR constraints, which were determined solely by the re-irradiation target anatomy and the estimated cumulative dose. The systemic therapy influenced only multidisciplinary sequencing (e.g., neoadjuvant ChT for downstaging before re-irradiation).

### 2.4. Follow-Up and Endpoints

Our institution receives patients from all over Spain as one of the leading PT centres in the country, as well as a few international patients, which presents a real challenge for standard follow-up. The protocol includes MRI and PET-CT at 3 months after PT completion, followed by clinical review at least every 6 months, either by in-person visit or structured remote contact, with extra imaging if symptoms are concerning. OS and PFS were measured from PT completion. Adverse events were graded with CTCAE v5.0 [23]. Those occurring within 90 days of treatment completion were classified as acute; events after 90 days were considered late.

### 2.5. Statistical Analysis

Statistical analyses were performed using R 4.4.0 (R Foundation for Statistical Computing, Vienna, Austria) with the survival and survminer packages. Continuous variables are reported as medians with ranges or interquartile ranges (IQR). Categorical variables are expressed as counts and percentages.

Three time-to-event outcomes were defined in this study. For OS, the event was death from any cause, counted from the last day of PT. PFS was defined as the first progression at any site or death, whichever occurred first. LRC was defined as the first locoregional recurrence and patients who died without locoregional failure were censored at the date of death, while those without events were censored at their last contact. Of the 65 re-irradiated patients, four were excluded from the time-to-event analyses because no follow-up or death date was recorded and they could therefore not contribute event or censoring information, leaving 61 evaluable patients. The same 61-patient cohort was used for all survival analyses (Kaplan–Meier estimates, log-rank comparisons and Cox models). Kaplan–Meier curves were generated for all three outcomes. Ninety-five percent confidence intervals were derived from Greenwood’s formula, and the median follow-up was determined using the reverse Kaplan–Meier method. The groups were compared using two-sided log-rank tests. The prespecified comparisons covered anatomical extent (central/skull base vs. peripheral), T-stage (T1–2 vs. T3–4) and N stage (N0 vs. N+) at re-irradiation, combined TNM group, treatment intent, concurrent chemotherapy, macroscopic disease and margin status in postoperative cases. As a sensitivity analysis for LRC, the cumulative incidence of locoregional failure was also estimated, treating death without locoregional failure as a competing event, and compared between anatomical groups using Gray’s test. Grade 3 or higher toxicity rates were compared between the anatomical groups using Fisher’s exact test. Continuous variables, including OAR doses and baseline characteristics, were compared between the anatomical groups using the Mann–Whitney U test. The significance threshold was set at *p* < 0.05. Cox proportional hazards models were used to assess OS, PFS and LRC. Variables with *p* < 0.10 in the univariate analysis, plus anatomical extent as a prespecified covariate, were entered into the multivariate models. The proportional hazards assumption was assessed using scaled Schoenfeld residuals (Supplementary Figure S1). It was satisfied for all covariates except T-stage in the PFS model (*p* = 0.046). The corresponding hazard ratio should therefore be interpreted as a time-averaged effect, consistent with the exploratory nature of the multivariable analyses. The numbers of events were: 25 deaths, 39 PFS events and 22 LRC events. All multivariate results were considered exploratory.

## 3. Results

### 3.1. Patient and Disease Characteristics

The cohort comprised 65 patients (Table 1). The patients’ ages ranged from 18 to 89 years, with a median of 60.6 years; 43 patients were men. The nasopharynx and the paranasal sinuses/nasal cavity each contributed 17 cases (26.2%), and were the two most common primary sites, followed by the oral cavity (*n* = 10, 15.4%) and oropharynx (*n* = 9, 13.8%). Squamous cell carcinoma accounted for approximately two-thirds of histologies (66.2%). Sixty patients had recurrent disease, and five had a second primary disease. ECOG performance status was 0 or 1 in 63 patients (96.9%) at the time of re-irradiation. Forty-five patients were treated with radical intent (69.2%) and 20 were treated postoperatively. Surgical margins in the postoperative group were R1 in 11 cases (55.0%), R0 in three (15.0%), R2 in three (15.0%) and unknown in three (15.0%).

Central/skull-base structures were involved in 45 patients (69.2%), and the rest had unilateral (16, 24.6%) or bilateral (4, 6.2%) disease. As the unilateral and bilateral cases were few and both lay outside the central compartment, we combined them into one peripheral category (20 patients, 30.8%) and compared it with the central/skull base group (45 patients, 69.2%).

Twelve patients (18.5%) received neoadjuvant chemotherapy before re-irradiation. Cisplatin-based regimens were used in six patients, and carboplatin was substituted in four patients due to contraindications or intolerance to cisplatin. The most frequent combination was cisplatin-gemcitabine (three patients), with a median of three neoadjuvant cycles (range 2–7). Concurrent chemotherapy was administered to 18 patients (27.7%): 12 in the radical group and six in the postoperative group. Weekly cisplatin was the most commonly used concurrent regimen (14 patients, 77.8%).

### 3.2. Primary Tumour Location and Recurrence Patterns

The nasopharynx and the area around the nasal cavity, including the paranasal sinuses, were the most common sites of tumour recurrence, with 17 cases in each location. In the paranasal sinuses, the maxillary and nasal cavities had an equal number of cases (six each), and the ethmoid bone had four cases. Tumours in the oropharynx were mostly found in the palatine tonsil (four cases) and at the base of the tongue (three cases). In the oral cavity, including the mouth, tumours were found in the tongue, retromolar trigone and gingiva or alveolar ridge (three cases each) (Table 2).

Same-site recurrence was common overall, but rates differed by primary location. It was 65% in the nasopharynx, 53% in the paranasal sinuses, and 50% in the oral cavity. Oropharyngeal tumours recurred at the same site in only 33% of cases, with the remaining recurrences appearing in the parotid gland, pharynx or masticator space. Laryngeal primaries showed a different pattern: cervical nodal disease was observed in two of five patients (40%), more often than recurrence at the glottis itself (1 of 5 patients, 20%).

In some cases, tumours did not recur in the same location but rather in anatomical areas near the original primary tumour. In our cohort, nasopharyngeal tumours tended to extend towards the cavernous sinus or pterygopalatine fossa, whereas paranasal sinus tumours reached the orbit or sphenoid bone. Tumours originating in the oral cavity also spread to the pterygopalatine fossa. These patterns of perineural and/or perivascular spread complicate the definition of CTV, making it necessary to delineate high-risk neuronal spread patterns.

### 3.3. Treatment Characteristics and Dosimetric Parameters

All patients were treated with the IMPT technique with a median prescribed dose of 66 Gy (RBE) in 30 fractions (2.2 Gy/fraction). Sixty (92.3%) patients completed treatment as planned. The five who did not complete it stopped because of toxicity or clinical deterioration.

A single CTV dose level was used in 69.2% of patients and two simultaneous dose levels in 30.8%. A single level was the most frequent approach in radical cases (71.1%), whereas two levels were more often needed in postoperative cases (35.0%), reflecting the requirement to cover both high-risk and intermediate-risk volumes after resection. High-risk and intermediate-risk CTVs were prescribed in 39 (60.0%) and 46 (70.8%) patients, respectively.

Coverage was adequate across both risk levels. For the high-risk CTV, the median D95 was 63.8 Gy (RBE), median D98 was 61.7 Gy (RBE), and median volume was 32.7 cc; the intermediate-risk CTV reached a median D95 of 58.0 Gy (RBE), a median D98 of 55.7 Gy (RBE) and a median volume of 66.0 cc (Table 3).

The dose distribution to OARs depended on the extent of the re-irradiated area (Table 4). In the central/skull-base group, the highest doses fell on the skull base and anterior cranial fossa structures: median brainstem Dmax was 16.4 Gy (RBE), optic chiasm Dmax was 17.1 Gy (RBE) (range 0–54) and optic nerve Dmax was 21.4 Gy (RBE) on the right and 26.7 Gy (RBE) on the left. The median temporal lobe Dmax was 48 Gy (RBE) on the left and 62.6 Gy (RBE) on the right in the patients with available data (*n* = 14–15). The brain Dmax reached a median of 68.5 Gy (RBE), yet the mean brain dose remained low (1.7 Gy (RBE)), reflecting the steep dose fall-off of PBS-IMPT. In the peripheral group, the dose distribution changed towards the structures in the lower part of the head and neck area. Here, the median mandible Dmax (61.5 Gy [RBE]), oral cavity Dmean (11.3 Gy [RBE]) and larynx Dmean (9.5 Gy [RBE]) all exceeded the corresponding central values (49.7, 4 and 0 Gy [RBE], respectively). The cervical spinal cord also received higher doses in this group, with a median Dmax of 3.8 vs. 0.4 Gy (RBE). In this group, the anterior optic structures, brainstem and temporal lobes did not receive much radiation. These inter-group differences were statistically significant for the brainstem, optic apparatus and brain (higher in central group) and for the mandible, oral cavity and larynx (higher in peripheral group); the spinal-cord difference showed only a trend (*p* = 0.061), and the temporal-lobe comparison was limited by the small number of peripheral cases with available data (Table 4).

Cumulative EQD2 doses, obtained by deformable accumulation of the prior and re-irradiation courses, could be reconstructed in 46 of the 65 patients (a usable prior dose distribution was available in 50) and the resulting doses to the principal OAR are summarised in Table 5. Given the deformable-registration uncertainty in this previously irradiated, anatomically altered region, these cumulative metrics should be interpreted with caution.

### 3.4. Oncological Outcomes

**Figure 3:**
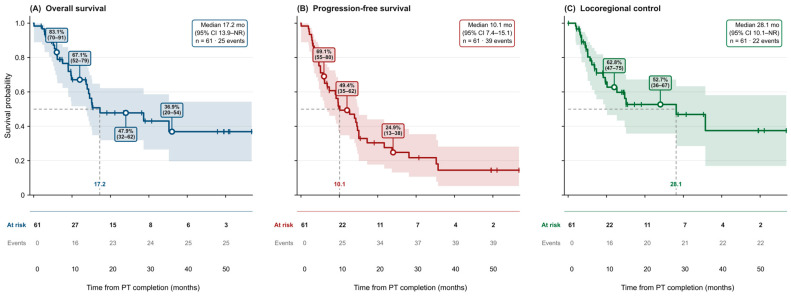
Kaplan–Meier curves for overall survival (**A**), progression-free survival (**B**) and locoregional control (**C**) in 58 evaluable patients. Shaded bands represent 95% confidence intervals.

Four patients with no recorded follow-up or death date were excluded from the survival analyses, leaving 61 evaluable cases (Supplementary Table S1). The median follow-up was 9.3 months (mean 14.7, range 0.1–56.8 months). Twenty-five patients died; the median OS was 17.2 months (95% CI 13.9–NR). OS at 6, 12, 24 and 36 months was 83.1%, 67.1%, 47.9% and 36.9%, respectively (Figure 3). Thirty-nine patients progressed. The median PFS was 10.1 months (95% CI 7.4–15.1), with rates of 69.1%, 49.4% and 24.9% at 6, 12 and 24 months, respectively. LRC was achieved for a longer duration: the median LRC was 28.1 months (95% CI 10.1–NR), with 12- and 24-month rates of 62.8% and 52.7%, respectively. In a competing-risks sensitivity analysis treating death as a competing event, the cumulative incidence of locoregional failure was 34.0% at 12 months and 41.2% at 24 months and did not differ significantly between central and peripheral disease (Gray’s test, *p* = 0.451), consistent with the primary analysis.

Twenty-six patients had confirmed disease progression. The site of failure was locoregional in most cases: six had purely local recurrence (23.1%), five had regional progression (19.2%) and seven had combined locoregional progression (26.9%), totalling 18 locoregional failures (69.2%). Distant metastases alone occurred in four patients (15.4%), and another four had concurrent locoregional and distant progression.

### 3.5. Outcomes Stratified by Anatomical Extent of Re-Irradiation

**Figure 4:**
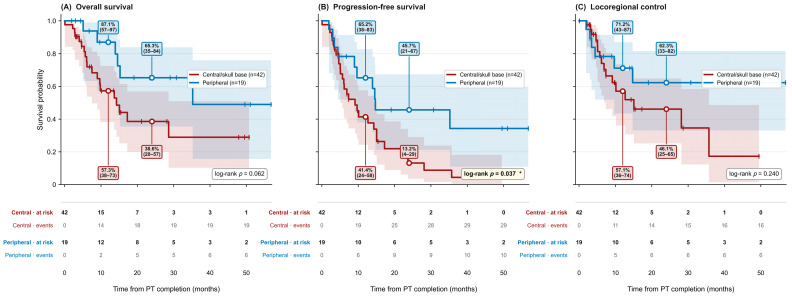
Kaplan–Meier curves by anatomical extent: central/skull base (red) vs. peripheral (blue). The panels show overall survival (**A**), progression-free survival (**B**) and locoregional control (**C**). * *p* < 0.05 (statistically significant).

Forty-five of the 65 patients had central/skull-base disease and 20 had peripheral disease; of these, 42 and 19, respectively, were evaluable for survival (Table 6). Kaplan–Meier analysis showed a non-significant trend toward worse OS in central disease (median 14.3 vs. 35.2 months for peripheral cases), with a 12-month rate of 57.3% vs. 87.1% (*p* = 0.062). PFS was significantly worse: 9.1 vs. 14.7 months (*p* = 0.037). LRC did not differ significantly between groups (median 15.1 months vs. not reached, *p* = 0.240). However, given the T-stage imbalance and the small number of events, this should not be interpreted as evidence of equivalence (Figure 4). The survival gap most likely reflects the case mix: the central compartment harboured more T3–T4 disease. A formal baseline comparison (Supplementary Table S2) confirmed that the groups were balanced for age, prior radiotherapy (number of courses, cumulative dose and interval), treatment intent and concurrent chemotherapy, differing significantly in the proportion of T3–T4 tumours (88.6% vs. 41.7%; *p* = 0.003), the only factor that retained prognostic significance on multivariable analysis. When T-stage was included in the Cox model, the anatomical location lost all prognostic weight (OS: HR 0.90, 95% CI 0.25–3.25, *p* = 0.871; PFS: HR 1.02, 95% CI 0.39–2.75, *p* = 0.962). Section 3.6 further develops this finding.

### 3.6. Clinical TNM Staging at Re-Irradiation and Impact on Survival

**Figure 5:**
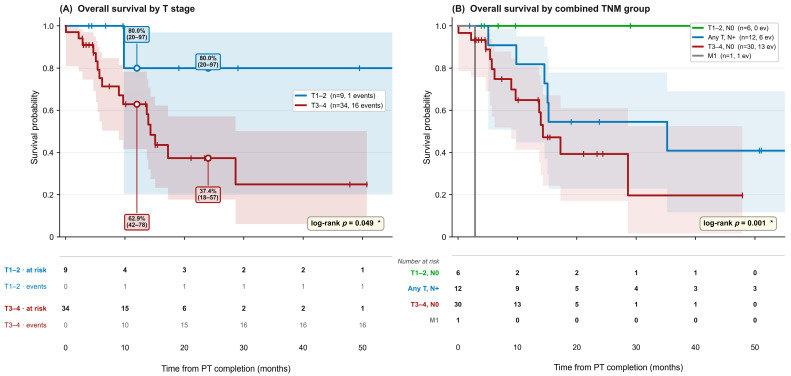
Overall survival by (**A**) T-stage (T1–T2 vs. T3–T4, log-rank *p* = 0.049) and (**B**) combined clinical group at re-irradiation (T1–T2 N0, any T N+, T3–T4 N0, M1; log-rank *p* = 0.001). * *p* < 0.05 (statistically significant).

The T category was recorded in 53 patients (81.5%). The distribution was heavily skewed towards T4 (32 patients, 60.4%), followed by T2 in eight (15.1%), T3 in four (7.5%) and T0, T1 or Tx in seven (13.2%). Nodal spread was documented in 13 patients (24.5%) and two (3.8%) had M1 disease at re-irradiation. T-stage clearly separated survival. Patients with T3–T4 disease had a median OS of 14.3 months; no deaths occurred among T1–T2 patients during follow-up, leaving the upper survival limit uncalculated. At 12 months, OS was 80% (95% CI 20–97) for T1–T2 vs. 62.8% (95% CI 41–78) for T3–T4 (log-rank *p* = 0.049; Figure 5A).

Adding nodal status produced four groups with distinct outcomes (Figure 5B). No deaths occurred in the T1–T2 N0 subgroup during the follow-up. Patients with nodal spread (any T, N+) had a 12-month OS of 81.8% (95% CI 36.5–93.9), which fell to 54.5% (95% CI 20.4–80.5) at 24 months. Those with T3–T4 N0 disease had a median OS of 14.3 months, with 12- and 24-month rates of 64.9% (95% CI 41.2–80.8) and 39.3% (95% CI 17–61), respectively. Node-positive patients appeared to have marginally better outcomes than T3–T4 N0 cases, which we tentatively attribute to greater eligibility for surgical salvage, although the numbers are too small for firm conclusions. M1 patients had the worst outcomes, with a median survival of 2.9 months. The four groups differed significantly (overall log-rank *p* = 0.001).

**Figure 6:**
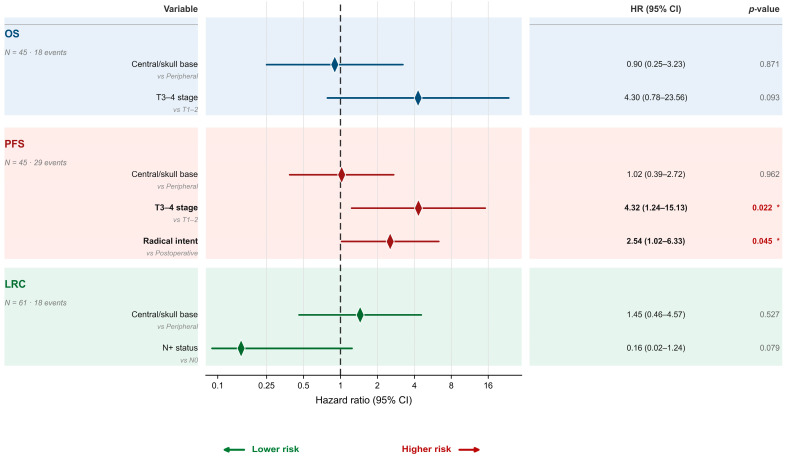
Multivariable Cox proportional hazards analysis for overall survival (OS, top, blue), progression-free survival (PFS, middle, red) and locoregional control (LRC, bottom, green). Forest plot of adjusted hazard ratios (HR) with 95% confidence intervals (CI); for each variable, the diamond marks the HR point estimate and the horizontal line its 95% CI, with the corresponding HR (95% CI) and *p*-value listed on the right. Each model includes only the variables selected for that outcome (*p* < 0.10 on univariable analysis plus anatomical extent as a pre-specified covariate). The vertical dashed line indicates HR = 1 (no effect): estimates to its left denote lower risk and to its right higher risk. The OS and PFS models include T-stage as a covariate and were therefore restricted to the 45 patients with an evaluable T category (complete-case analysis), whereas the LRC model included nodal status rather than T-stage and comprised all 61 evaluable patients. Statistically significant associations (*p* < 0.05) are highlighted with an asterisk and bold red *p*-values. HR, hazard ratio; CI, confidence interval.

Multivariate Cox regression analysis revealed that T3–T4 stage was the only factor retaining significance for PFS (HR 4.32, 95% CI 1.24–15.13; *p* = 0.022), with a non-significant trend in the same direction for OS (HR 4.30, 95% CI 0.78–23.53; *p* = 0.093). Radical intent also reached significance for PFS compared with postoperative re-irradiation (HR 2.54, 95% CI 1.02–6.33; *p* = 0.045). This most likely reflects confounding by indication, as radical patients had unresectable gross disease and were not a comparable population. Once T-stage was entered into the model, anatomical location lost all prognostic weight (PFS: HR 1.02, 95% CI 0.39–2.72; *p* = 0.962), consistent with the view that the Kaplan–Meier survival gap in central cases reflects case mix rather than anatomical location per se. The time intervals from prior radiotherapy, nodal status and concurrent chemotherapy were non-significant for every endpoint. With 9.0–9.7 events per variable, these results are hypothesis-generating (Figure 6).

### 3.7. Impact of Time Interval Between Prior Radiotherapy and Re-Irradiation

The median interval between prior RT and PT was 34 months (range, 11–300; IQR, 23–92). To better understand how time without disease affects outcomes, we performed additional analyses using a 24-month cutoff, as suggested by the MIRI Collaborative [9], and used the cohort median of 36 months to determine if the results were consistent. The interval was assessable in all 65 patients; 58 of them had complete time-to-event data and were included in the survival analysis.

At the 24-month cutoff, 17 patients had a short interval (≤24 months) and 41 had a longer one. The median OS was 15.2 months in the short-interval group vs. 28.6 months in the long-interval group. The 24-month OS rates were 31.9% vs. 54.6%, a difference that did not reach statistical significance. PFS showed the same pattern: 15.1 vs. 28.1 months, also without significance (Table 7).

A sensitivity analysis at the 36-month cohort median (*n* = 29 vs. 29) showed a median OS of 15.1 vs. 28.6 months (24-month OS 31.3% vs. 59.3%; *p* = 0.448), median PFS of 9.7 vs. 10.1 months (*p* = 0.554), and median LRC of 15.1 vs. 28.1 months (*p* = 0.542). The direction was consistent for both cutoffs. Longer intervals tracked better 24-month OS at each threshold, but no endpoint was significant.

### 3.8. Adverse Events

We collected adverse event data according to CTCAE v5.0 [23], grouping each event into G1–G2 versus ≥G3 and categorising them by the anatomical extent of the re-irradiated volume. We chose this method because the dosimetric profiles of the central/skull base and peripheral targets are notably different, and the patterns of acute and late events closely follow this difference.

#### 3.8.1. Acute Adverse Events

Radiodermatitis was the most frequent acute event; 55 of 65 patients (84.6%) had any grade, but 53 were G1–G2 and only two reached ≥ G3 (3.1%). Oral mucositis affected 33 patients (50.8%), with five ≥ G3 events (7.7%) (Table 8). Fatigue occurred in about a third (32.3%; all G1–G2). Other acute events, including odynophagia, pharyngeal mucositis, dysphagia, pain, ocular events, oesophagitis and nasosinusitis, each appeared in fewer than 10% of the patients. The anatomical split was clinically coherent. G3 or higher oral mucositis was more common peripherally than centrally (4 of 20, 20.0% vs. 1 of 45, 2.2%; *p* = 0.028), and ≥ G3 radiodermatitis showed the same tendency without reaching significance (10.0% vs. 0%; *p* = 0.091). This corresponds to the larger mucosal and skin doses in peripheral cases (oral cavity Dmean 11.3 vs. 4.0 Gy; mandibular Dmax 61.5 vs. 49.7 Gy). Fatigue was more frequent centrally (40.0% vs. 15.0%; *p* = 0.083). Four patients (6.2%) needed percutaneous endoscopic gastrostomy (PEG), all in the central group (three prophylactic, one toxicity-driven). Fisher’s test was not significant (*p* = 0.303). No grade 4 or 5 acute events were recorded in either group.

#### 3.8.2. Late Adverse Events

The interpretation of late events requires caution. The median follow-up was short (9.3 months), and late data could not be recorded for a substantial proportion of patients, either because they had not yet reached the late assessment window or because they were followed up at distant referring centres. Furthermore, serious late complications such as carotid blowout, temporal lobe necrosis, cranial neuropathy and osteoradionecrosis may not manifest for several years and will be captured only with extended follow-up. Across the whole cohort, late dysphagia was the most frequent finding (10 patients, 15.4%), with nine G1–G2 and one ≥ G3 event(s). Late xerostomia followed (nine patients, 13.8%), all G1–G2. Osteoradionecrosis occurred in four patients (6.2%; all G1–G2). Late dysarthria was recorded in four patients (6.2%; all G1–G2). Late feeding-tube dependence and late fistula occurred in one patient each (1.5%), both in peripheral cases. We did not record any episode of late bleeding. Two late neurological events were observed (both G1–G2), with one trigeminal neuropathy and one hypoglossal palsy. The only late event that approached statistical significance between the groups was late dysphagia, which was more frequent in peripheral cases (30.0%) than in central cases (8.9%; *p* = 0.057) (Table 9). The proximity of peripheral targets to the pharyngeal constrictors, larynx and proximal oesophagus is a plausible explanation. All other late events were distributed evenly between the groups. No treatment-related grade 5 late events were recorded during the observation period.

## 4. Discussion

Three points emerged from this 65-patient cohort. The survival figures were comparable to those reported in the largest published proton re-irradiation series. Acute adverse events were well below those typically associated with photon-based re-irradiation, despite a median dose of 66 Gy delivered to a heavily pretreated field. T3–T4 tumour stage at re-irradiation proved to be the strongest predictor of shorter PFS; the apparent prognostic weight of central/skull-base anatomy observed on Kaplan–Meier curves disappeared once T-stage was included in the multivariable model. To our knowledge, this is one of the largest contemporary European single-centre series of PBS-IMPT re-irradiation specifically reported, given that most prior European experience has been published with passive scattering, carbon ions or pooled IMRT/proton cohorts [17,19,24].

Our 17.2-month median OS and 65.5% 12-month OS are within the range published by the largest proton cohorts. McDonald et al. reported 16.5 months in 61 patients, Romesser et al. reported 65.2% at 12 months in 92 patients, and Phan reported. approximately 70% 2-year OS at MD Anderson in 60 patients [12,13,14]. The recent 242-patient series by Lee et al. [15] provides the closest comparison with 66.6% at 12 months, which is almost the same proportion we observed. The technique used is what differentiates our cohort: we treated everyone with PBS, while most proton re-irradiation series used conventional proton beam delivery [12,14]. Beddok et al. found broadly comparable survival and a marked predominance of in-field or marginal failure in 55 patients re-irradiated with IMRT or protons, which is the same pattern we noted [24]. To place all these findings in a photon context, the MIRI Collaborative, with 412 IMRT-treated patients, reported 2-year OS rates of 17–62% depending on the recursive partitioning class [9].

We applied the MIRI recursive partitioning classes retrospectively to our cohort [9]. Twelve patients (20%) met the Class I criteria (prior surgical resection with a re-irradiation interval exceeding 2 years), while the remaining 48 (80%) fell into Class II; none qualified for Class III, which the MIRI group defined as a short interval combined with pretreatment feeding-tube or tracheostomy dependence. This distribution is not a data artefact, as virtually all our patients scored ECOG 0–1 at the start of re-irradiation, and none were dependent on enteral feeding beforehand, which is consistent with the tighter patient-selection criteria that are typically applied in proton programmes. Our 12-month OS of 83% in Class I and 62% in Class II both exceed the MIRI 2-year benchmarks of 61.9% and 40.0%, respectively, for those same strata. The contrast is instructive on two counts. PBS-IMPT could improve survival data in this well-filtered population, but the near-absence of Class III patients in our series means we currently have no data on whether its favourable toxicity profile applies to patients with pre-existing organ dysfunction, who are excluded from most proton programmes.

The most striking aspect of these results was the acute toxicity profile. Only 7.7% of patients had grade ≥3 oral mucositis, and that was in a cohort where 86.2% started treatment with gross residual disease and 63.1% had central or skull-base recurrence. The 20–40% figure usually quoted for IMRT re-irradiation is far above what we observed, and our numbers fit the pattern reported in dedicated proton cohorts [6,7,8,17,25]. We consider that this is mostly due to the dosimetric profile of PBS-IMPT and the robust optimisation performed on the CTV, although patient selection probably played some role [20,26]. A recent dosimetric study by Beddok et al. identified the pharyngeal constrictor muscles and the oral cavity as the most crucial structures for late functional outcomes after re-irradiation [27]. Adding those constraints to future PBS-IMPT planning protocols, alongside the brainstem, optic apparatus and carotid limits we already use, looks like a reasonable step.

Late toxicity is difficult to interpret given the short follow-up duration (median, 9.3 months) and the fact that only some of the cohort had enough time for delayed effects to appear. Considering this limitation, the 6.2% osteoradionecrosis rate (all G1–G2) is comparable to the 5–17% range reported in photon series [6,7,8,21,27]. Although median temporal lobe Dmax in the re-irradiation course reached approximately 61 Gy in central cases, no temporal lobe necrosis or brainstem injury was observed during this short follow-up period. However, these late neurological complications could appear with longer follow-up. Functional issues, such as feeding-tube or tracheostomy dependency, are now considered a key part of the patient experience after re-irradiation, and they require their own structured assessment once our follow-up is extended [28]. No treatment-related grade 5 events occurred during the current observation period [11,22,26]. Cumulative EQD2 doses for several critical structures showed medians close to 110 Gy for the carotid arteries, around 99 Gy for the mandible, near 80 Gy for the brain and temporal lobes, 45 Gy for the brainstem and 33 Gy for spinal cord (Table 5). Even at these levels, severe late events remained uncommon, which suggests that PBS-IMPT re-irradiation may be feasible when planning is guided by individualised cumulative-dose estimates, in line with published cumulative dose–toxicity data, although our short follow-up means this observation should be viewed cautiously [21].

The central/skull base versus peripheral split is, for us, the most directly actionable finding in our study. Central tumours exhibited worse OS (median 14.3 months vs. not reached; *p* = 0.040) and PFS (9.1 vs. 14.7 months; *p* = 0.045), yet the LRC rates were essentially the same in both groups (*p* = 0.349). On multivariable Cox regression, once T-stage was entered into the model, anatomical location provided no additional prognostic weight (PFS HR 1.04, 95% CI 0.39–2.75; *p* = 0.941). The excess mortality in central/skull-base cases thus reflects the disproportionate share of T3–T4 tumours treated in that compartment, and is not an inherent effect of the site. PBS-IMPT controls local disease similarly regardless of where the tumour is situated; when progression occurs close to the brainstem, optic pathway or carotid, both the dose that can be given and the salvage options available notably reduce. The pattern of failure supports this notion. Locoregional events accounted for 69.2% of all progressions; isolated distant failure was uncommon (15.4%), in line with the literature [9,24]. A noticeable share of cases of recurrence in our study were associated with perineural and perivascular routes (pterygopalatine fossa, cavernous sinus and masticator space), especially in cases of nasopharyngeal and adenoid cystic carcinoma. This argues for explicitly considering these high-risk perineural pathways in the CTV, as the MIRI Collaborative has already stated for photon modalities; Beddok et al. also reinforced this notion in their patterns-of-failure analysis with IMRT and protons [9,24].

The anatomical pattern of failure in our cohort offers a clue to the underlying mechanism of recurrence. The predominance of in-field, locoregional failure (69.2% of progressions), as observed in other series on re-irradiation with photons and protons [19,29,30], suggests that most recurrences reflect intrinsic radioresistance (taking into account hypoxia, high clonogenic density and accelerated repopulation) rather than geographic miss. Whereas geographic miss can be corrected through better delineation, radioresistant disease tends to recur repeatedly within the same subvolume despite adequate dose. Functional and molecular imaging with FDG-PET, hypoxia tracers such as ^18^F-FMISO, and diffusion-weighted/functional MRI have been proposed to localise these resistant subvolumes for biologically guided dose escalation, and a randomised phase 2 trial of adaptive FDG-PET dose-painting by numbers improved local control in head and neck cancer [31]. This strategy remains investigational since hypoxia is dynamic and recurrences do not consistently arise within the pre-treatment hypoxic volume [32]; nonetheless, integrating functional imaging into PBS-IMPT re-irradiation planning is an attractive route toward more personalised, resistance-adapted target definition.

The use of concurrent systemic therapy in the re-irradiation setting remains an unresolved question. The available evidence does not currently support routine concurrent chemotherapy in HNC re-irradiation, and the heterogeneity of indications, regimens and patient selection across published cohorts complicates any between-series comparisons [22,33]. In the future, the systemic side of the equation is more likely to evolve through immune checkpoint inhibitors rather than through more aggressive cytotoxic regimens [34].

T-stage at re-irradiation was a more critical determinant of outcomes than any other variable in our study. T3–T4 disease was associated with shorter OS on Kaplan–Meier analysis (*p* = 0.048) and was the sole covariate retaining significance for PFS in the multivariable model (HR 4.32, 95% CI 1.24–15.13; *p* = 0.022). Moreover, the combined staging analysis (T1–2 N0, Any T N+, T3–4 N0, M1; *p* = 0.001) provided the most distinct categorisation across the dataset, with no deaths in the T1–2 N0 group and uniformly poor outcomes in M1. The time interval between prior radiotherapy and re-irradiation failed to reach significance in either the univariate or multivariate models in contrast with some IMRT series [5,9], despite numerically inferior outcomes in patients treated within 24 months. Furthermore, other series also found no disease-free interval as a major prognostic factor [35]. With only 17 patients in the short-interval group, the cohort was not sufficiently powered to detect a meaningful effect and selection pressure at a high-volume national referral centre further compounded this issue. Standardised reporting of TNM stage at re-irradiation, disease-free interval and anatomical extent would be a practical step forward, and future series could at least be compared systematically even when none is individually large enough to identify dominant prognostic factors.

Several other analyses are ongoing at our centre and will be presented separately. The first aims to quantify how much of the prior planning target volume overlaps with the current re-irradiation target, because dosimetric overlap is a consistent predictor of late toxicity in this setting [21,22]. The second aims to determine absolute lymphocyte counts at baseline and at the end of treatment in order to specifically describe radiation-induced lymphopenia under PBS-IMPT [36]. The third aims to estimate the effective dose to circulating immune cells (EDIC) using the framework first proposed for thoracic radiotherapy and recently extended to head and neck targets, with an integral-dose-based model as the working surrogate [37,38,39,40,41]. It is biologically reasonable to determine if PBS-IMPT shifts the relationship between integral dose and immune-cell exposure compared with photon modalities, and the answer matters when re-irradiation is combined with immune checkpoint inhibitors [34].

This study has important limitations. This was a single-centre retrospective analysis of a prospectively maintained registry, and the patient profile has the inevitable selection biases of a national referral centre. Our median follow-up of 9.3 months is well below the 18–24 months that is usually quoted as the minimum for proper late toxicity assessment; therefore, the late toxicity figures we report are preliminary by definition and will increase with longer observation [27,42]. Cumulative dose reconstruction was feasible in 46 of the 65 patients, which precluded formal OAR dose–response analyses across the whole cohort; extending this to all patients is an aim of ongoing work [22,35]. The cohort was also heterogeneous in primary site, histology and treatment intent, which is normal for re-irradiation referrals; however, this limited the power of any subgroup analysis. Without a matched photon comparator, we could not directly determine if PBS-IMPT outperforms modern IMRT in this scenario [26]. Our work going forward is straightforward: extend follow-up; complete the overlap, lymphopenia and EDIC analyses outlined above; utilise deformable image registration in our workflow to track cumulative OAR doses across radiotherapy courses; incorporate validated patient-reported outcome instruments such EORTC QLQ-C30/HN35 and the MD Anderson Dysphagia Inventory; and provide multi-institutional prospective registries with patient-level data.

## 5. Conclusions

Re-irradiation of recurrent HNC with PBS-IMPT should not be an experimental salvage option reserved for a few patients. In our experience, it has become a viable treatment option for those with previously irradiated tumours, provided that planning is robust and cumulative-dose limits are respected. The favourable toxicity profile we observed was predominantly acute and given the short median follow-up (9.3 months), estimates of late toxicity remain preliminary and will require longer observation before definitive conclusions can be drawn. T-stage at re-irradiation independently predicted shorter PFS and explained most of the poor survival outcomes observed in central/skull-base cases on Kaplan–Meier analysis, a difference that largely reflected the heavier T3–T4 burden in that compartment, since LRC did not differ significantly across anatomical sites. Patients with T3–T4 disease or unresectable tumours requiring radical re-irradiation need careful pretreatment counselling and should be prioritised for enrolment in prospective trials. The questions that remain will not be answered by single centres: late toxicity, the immune cost of treatment, and the actual impact of cumulative dose. Longer follow-up is required for the immunological and dosimetric parameters that we and other researchers are beginning to collect and to pool patients from different institutions in prospective collaborations.

## Figures and Tables

**Table 1:** Patient and disease characteristics (*n* = 65).

Characteristic	*n* (%)
**Age (years), median (range)**	60.6 (18.2–88.8)
**Sex**	
Male	43 (66.2%)
Female	22 (33.8%)
**Primary tumour site**	
Nasopharynx	17 (26.2%)
Paranasal sinuses/Nasal cavity	17 (26.2%)
Oral cavity	10 (15.4%)
Oropharynx	9 (13.8%)
Larynx	5 (7.7%)
Other (lacrimal gland, ear, salivary, TUO)	7 (10.8%)
**Histology at re-RT**	
Squamous cell carcinoma	44 (67.7%)
Adenoid cystic carcinoma	8 (12.3%)
Esthesioneuroblastoma	3 (4.6%)
Adenocarcinoma/ITAC	4 (6.2%)
Other	6 (9.2%)
**Disease setting**	
Recurrent disease	60 (92.3%)
Second primary	5 (7.7%)
**Number of prior RT courses**	
1	55 (84.6%)
2	9 (13.8%)
3	1 (1.5%)
**Prior RT dose (Gy), median (range)**	66 (24–80.5)
**Prior RT modality: VMAT/IMRT**	61 (93.8%)
**Interval prior RT to PT (months), median (range)**	34 (11–300)
**Treatment intent**	
Radical	45 (69.2%)
Postoperative	20 (30.8%)
Margin status	
R0	3 (15%)
R1	11 (55%)
R2	3 (15%)
Unknown	3 (15%)
**ECOG at PT**	
0	53 (81.5%)
1	10 (15.4%)
2	2 (3.1%)
**Gross disease at PT treatment**	56 (86.2%)
**Anatomical extent of re-irradiation**	
Central/skull base	45 (69.2%)
Unilateral	16 (24.6%)
Bilateral	4 (6.2%)
**Neoadjuvant ChT**	12 (18.5%)
Cisplatin-based regimens	6 (50%)
Carboplatin-based regimens	4 (33.3%)
Other	2 (16.7%)
**Concurrent ChT**	18 (27.7%)
Weekly cisplatin	14 (77.8%)
Weekly carboplatin	1 (5.6%)
Others	3 (16.6%)

Bold text denotes the variable (characteristic) headings and the non-bold rows listed beneath each heading are its categories. Re-RT: re-irradiation; TUO: tumour of unknown origin; ITAC: intestinal-type adenocarcinoma; RT: radiotherapy; PT: proton therapy; VMAT: volumetric-modulated arc therapy; IMRT: intensity-modulated radiation therapy; ChT: chemotherapy.

**Table 2:** Primary tumour location and corresponding recurrence sites at the time of re-irradiation.

Primary Location (*n*)	Primary Sublocations	Recurrence Sites at Re-Irradiation	Same-Site (%)
Nasopharynx (*n* = 17)	Nasopharynx (*n* = 17)	Nasopharynx (*n* = 11), cervical LN (*n* = 1), parotid (*n* = 1), maxillary sinus (*n* = 1), cavernous sinus (*n* = 1), pterygopalatine (*n* = 1), ethmoid (*n* = 1)	65%
Paranasal/Nasal (*n* = 17)	Maxillary sinus (*n* = 6), nasal cavity (*n* = 6), ethmoid (*n* = 4), frontal (*n* = 1)	Maxillary sinus (*n* = 5), nasal cavity (*n* = 4), orbit (*n* = 2), sphenoid (*n* = 2), ethmoid (*n* = 1), cervical LN (*n* = 1), nasopharynx (*n* = 1), pterygopalatine (*n* = 1)	53%
Oral cavity (*n* = 10)	Tongue (*n* = 3), retromolar (*n* = 3), gum (*n* = 2), alveolar ridge (*n* = 1)	Tongue (*n* = 3), retromolar (*n* = 3), gum (*n* = 1), pterygopalatine (*n* = 1), palatine tonsil (*n* = 1), cervical LN (*n* = 1)	50%
Oropharynx (*n* = 9)	Palatine tonsil (*n* = 4), base of tongue (*n* = 3), soft palate (*n* = 2)	Base of tongue (*n* = 3), parotid (*n* = 1), post. pharyngeal wall (*n* = 1), pharynx (*n* = 1), nasal cavity (*n* = 1), ethmoid (*n* = 1), masticatory space (*n* = 1)	33%
Larynx (*n* = 5)	Glottis (*n* = 3), supraglottis (*n* = 2)	Cervical LN (*n* = 2), palatine tonsil (*n* = 1), pharynx (*n* = 1), glottis (*n* = 1)	20%

Same-site recurrence was defined as recurrence within the same anatomical subsite as the primary tumour. LN: lymph node.

**Table 3:** Dosimetric parameters by CTV risk level and treatment intent.

CTV Level	Intent	*n*	Volume (cc)	D95 (Gy (RBE))	D98 (Gy (RBE))	D50 (Gy (RBE))	Dmean (Gy (RBE))
High Risk	All	39	32.7 (2.3–339.2)	63.8 (52.9–70.5)	61.7 (39.3–70.2)	67.5 (64.2–72.9)	67.1 (63.9–72.2)
	Radical	27	32.8 (6.5–149.4)	64.1 (52.9–70.5)	61.7 (44.2–70.2)	67.5 (66.4–72.9)	67.3 (64.8–72.2)
	Postop.	12	26.3 (2.3–339.2)	63.7 (57.4–69.2)	62.6 (39.3–68.9)	67.3 (64.2–70.2)	66.9 (63.9–70.3)
Intermediate Risk	All	46	66.0 (8.3–348.2)	58.0 (0–66.4)	55.7 (0–65.8)	61.9 (0–71.2)	61.8 (0–68.8)
	Radical	31	63.1 (8.3–344.1)	58.5 (44.3–66.4)	56.1 (38.6–65.8)	62.3 (46.5–71.2)	62.2 (46.5–68.8)
	Postop.	15	74.2 (14.8–348.2)	57.0 (0–61.0)	53.4 (0–59.4)	61.8 (0–67.6)	61.5 (0–66.7)

Data are presented as medians (range). Postop.: postoperative.

**Table 4:** Dose to organs at risk during PT re-irradiation, stratified by anatomical extent of the re-irradiated volume.

Organ at Risk	Central/Skull Base	Peripheral	*p*
Brainstem, D_max_	16.4 (0–61)	0.6 (0–14)	**0.001**
Optic chiasm, D_max_	17.1 (0–54)	0.0 (0–0)	**<0.001**
Optic nerve, right, D_max_	21.4 (0–71)	0.1 (0–10)	**<0.001**
Optic nerve, left, D_max_	26.7 (0–63)	0.1 (0–14)	**<0.001**
Temporal lobe, right, D_max_	62.6 (0–75)	NA †	0.133
Temporal lobe, left, D_max_	48.0 (4–77)	NA †	1.000
Brain, D_max_	68.5 (0–77)	9.7 (0–70)	**<0.001**
Brain, D_mean_	1.7 (0–8)	0.0 (0–3)	**<0.001**
Mandible, D_max_	49.7 (0–70)	61.5 (30–72)	**0.029**
Oral cavity, D_mean_	4.0 (0–45)	11.3 (0–56)	**0.011**
Larynx, D_mean_	0.0 (0–47)	9.5 (0–56)	**<0.001**
Spinal cord, D_max_	0.4 (0–29)	3.8 (0–16)	0.061

Data are median (range) in Gy (RBE). *p*-values from Mann–Whitney U test. Bold *p*-values indicate statistical significance (*p* < 0.05). † Too few peripheral cases with available data (*n* = 1) for meaningful comparison. D_max_, maximum dose; D_mean_, mean dose; NA, not applicable; RBE, relative biological effectiveness.

**Table 5:** Cumulative EQD2 doses to the principal OAR (prior and re-irradiation courses).

Organ at Risk	Cumulative EQD2 (Gy), Median (Range)	*n*
Brainstem, D_max_	45.3 (0.5–84.8)	44
Spinal cord, D_max_	32.8 (0–88.8)	45
Optic chiasm, D_max_	29.9 (0.7–67.4)	36
Optic nerve, right, D_max_	25.1 (0–90.0)	44
Optic nerve, left, D_max_	25.9 (0–96.3)	44
Brain, D_max_	78.6 (0.4–140.8)	39
Brain, D_mean_	5.9 (0.1–22.5)	39
Temporal lobe, right, D_max_	86.7 (22.5–116.0)	6
Temporal lobe, left, D_max_	74.8 (59.9–91.1)	6
Mandible, D_max_	99.3 (12.3–134.3)	45
Oral cavity, D_mean_	42.1 (3.7–110.1)	44
Larynx, D_mean_	40.3 (0–125.7)	41
Carotid artery, right, D_max_	115.0 (69.2–125.1)	7
Carotid artery, left, D_max_	109.5 (59.6–120.3)	8

Data are median (range) in Gy, expressed as the cumulative EQD2 dose (α/β = 2 Gy for late-responding tissues) summing the deformably registered prior course and the proton re-irradiation plan. The number of patients (*n*) varies by organ because some structures were only accumulated where anatomically relevant. D_max_, maximum dose; D_mean_, mean dose.

**Table 6:** Outcomes by anatomical extent of re-irradiation.

Oncological Outcomes	Central/Skull Base (*n* = 42 Evaluable)	Peripheral (*n* = 19 Evaluable)	*p*-Value
Median OS (months)	14.3	35.2	0.062
12-month OS	57.3%	87.1%	
24-month OS	38.6%	65.3%	
Median PFS (months)	9.1	14.7	0.037 *
Median LRC (months)	15.1	Not reached	0.240
Deaths	19/42 (45.2%)	6/19 (31.6%)	

Log-rank test for OS, PFS and LRC. * *p* < 0.05 (statistically significant).

**Table 7:** Outcomes by interval from prior RT to proton re-irradiation (24-month cutoff).

Endpoint	≤24 Months (*n* = 17)	>24 Months (*n* = 41)	*p*-Value
Median OS, months	15.2	28.6	0.780
12-month OS, %	70.9	63.0	—
24-month OS, %	31.9	54.6	—
Median PFS, months	13.9	9.7	0.662
12-month PFS, %	52.3	44.5	—
24-month PFS, %	22.4	23.9	—
Median LRC, months	15.1	28.1	0.926
12-month LRC, %	59.8	60.7	—
24-month LRC, %	47.8	50.6	—

**Table 8:** Acute adverse events.

Adverse Event	Total (*n* = 65)	Central/Skull Base (*n* = 45)		Peripheral (*n* = 20)		
		G1–G2	≥G3	G1–G2	≥G3	*p* (≥G3)
Radiodermatitis	55 (84.6%)	36 (80%)	0	17 (85%)	2 (10%)	0.091
Oral mucositis	33 (50.8%)	18 (40%)	1 (2.2%)	10 (50%)	4 (20%)	0.028 *
Fatigue/asthenia	21 (32.3%)	18 (40%)	0 (0%)	3 (15%)	0 (0%)	—
Odynophagia	5 (7.7%)	2 (4.4%)	0 (0%)	3 (15%)	0 (0%)	—
Pharyngeal mucositis	4 (6.2%)	0 (0%)	1 (2.2%)	3 (15%)	0 (0%)	—
Dysphagia	4 (6.2%)	3 (6.6%)	0 (0%)	1 (5%)	0 (0%)	—
Pain	3 (4.6%)	2 (4.4%)	0 (0%)	1 (5%)	0 (0%)	—
Blepharoconjunctivitis	3 (4.6%)	3 (6.6%)	0 (0%)	0 (0%)	0 (0%)	—
Nasosinusitis	3 (4.6%)	3 (6.6%)	0 (0%)	0 (0%)	0 (0%)	—
Corneal ulcer	2 (3.1%)	0 (0%)	2 (4.4%)	0 (0%)	0 (0%)	—
Esophagitis	1 (1.5%)	0 (0%)	0 (0%)	1 (5%)	0 (0%)	—
PEG tube placement	4 (6.2%)	4 (8.8%)	0 (0%)	0 (0%)	0 (0%)	—

*p*-values from Fisher’s exact test comparing ≥ G3 rates between anatomical groups; * *p* < 0.05 (statistically significant). PEG: percutaneous endoscopic gastrostomy.

**Table 9:** Late adverse events.

Adverse 65	Total (*n* = 65)	Central/Skull Base (*n* = 45)		Peripheral (*n* = 20)		
		G1–G2	≥G3	G1–G2	≥G3	*p* (Any Grade)
Late dysphagia	10 (15.4%)	4 (8.9%)	0 (0%)	5 (25.0%)	1 (5.0%)	0.057 *
Late xerostomia	9 (13.8%)	5 (11.1%)	0 (0%)	4 (20.0%)	0 (0%)	0.440
Osteoradionecrosis	4 (6.2%)	2 (4.4%)	0 (0%)	2 (10.0%)	0 (0%)	0.581
Late dysarthria	4 (6.2%)	2 (4.4%)	0 (0%)	2 (10.0%)	0 (0%)	0.581
Late feeding-tube dependence	1 (1.5%)	0 (0%)	0 (0%)	1 (5.0%)	0 (0%)	0.308
Late fistula	1 (1.5%)	0 (0%)	0 (0%)	1 (5.0%)	0 (0%)	0.308
Late bleeding	0 (0%)	0 (0%)	0 (0%)	0 (0%)	0 (0%)	1.000
Late neurological event	2 (3.1%)	0 (0%)	0 (0%)	2 (10.0%)	0 (0%)	0.091

Data are presented as *n* (% of the whole cohort). *p*-values from Fisher’s exact test for any-grade rates between the anatomical groups. * *p* < 0.10.

## Data Availability

The data presented in this study are available on request from the corresponding author. The data are not publicly available due to patient privacy.

## Supplementary Materials

The following supporting information can be downloaded at: https://www.mdpi.com/article/10.3390/cancers18142207/s1, Figure S1: Scaled Schoenfeld residuals for the multivariable Cox proportional hazards models; Table S1: Patients included, events and censored observations for each time-to-event endpoint; Table S2: Baseline characteristics by anatomical extent of re-irradiation.

## Abbreviations

The following abbreviations are used in this manuscript:

BED	Biologically effective dose
ChT	Chemotherapy
CI	Confidence interval
CTCAE	Common Terminology Criteria for Adverse Events
CTV	Clinical target volume
D50/D95/D98	Dose covering 50%/95%/98% of target volume
Dmean	Mean dose
Dmax	Maximum dose
ECOG	Eastern Cooperative Oncology Group
EDIC	Effective Dose to Immune Cells
EQD2	Equivalent dose in 2 Gy fractions
GTV	Gross tumour volume
HNC	Head and neck cancer
IMPT	Intensity-modulated proton therapy
IMRT	Intensity-modulated radiation therapy
IQR	Interquartile range
ITAC	Intestinal-type adenocarcinoma
LN	Lymph nodes
LRC	Locoregional control
M1	Distant metastasis
MIRI	Multi-Institution Re-irradiation
MRI	Magnetic resonance imaging
MSK	Memorial Sloan Kettering
N+	Lymph node positive
OAR	Organs at risk
OS	Overall survival
PBS	Pencil-beam scanning
PBS-IMPT	Pencil-beam scanning intensity-modulated proton therapy
PEG	Percutaneous endoscopic gastrostomy
PET-CT	Positron emission tomography–computed tomography
PFS	Progression-free survival
PT	Proton therapy
PTV	Planning target volume
R0/R1/R2	Resection margin status (complete/microscopic/macroscopic)
RBE	Relative biological effectiveness
RS	Range shifter
SBRT	Stereotactic body radiation therapy
TNM	Tumour-node-metastasis
TPS	Treatment planning system
TUO	Tumour of unknown origin
VMAT	Volumetric modulated arc therapy
