# Supplementary material for: Salvage Proton Therapy Re-Irradiation in Recurrent Head and Neck Cancer: Outcomes and Adverse Events by Re-Irradiated Target Site
Source: Cancers (Basel). 2026 Jul 9;18(14):2207. doi: 10.3390/cancers18142207 (PMC13406275; doi:10.3390/cancers18142207)
Supplement: Supplementary file 1 [file cancers-18-02207-s001.zip › cancers-4366375-supplementary.pdf]

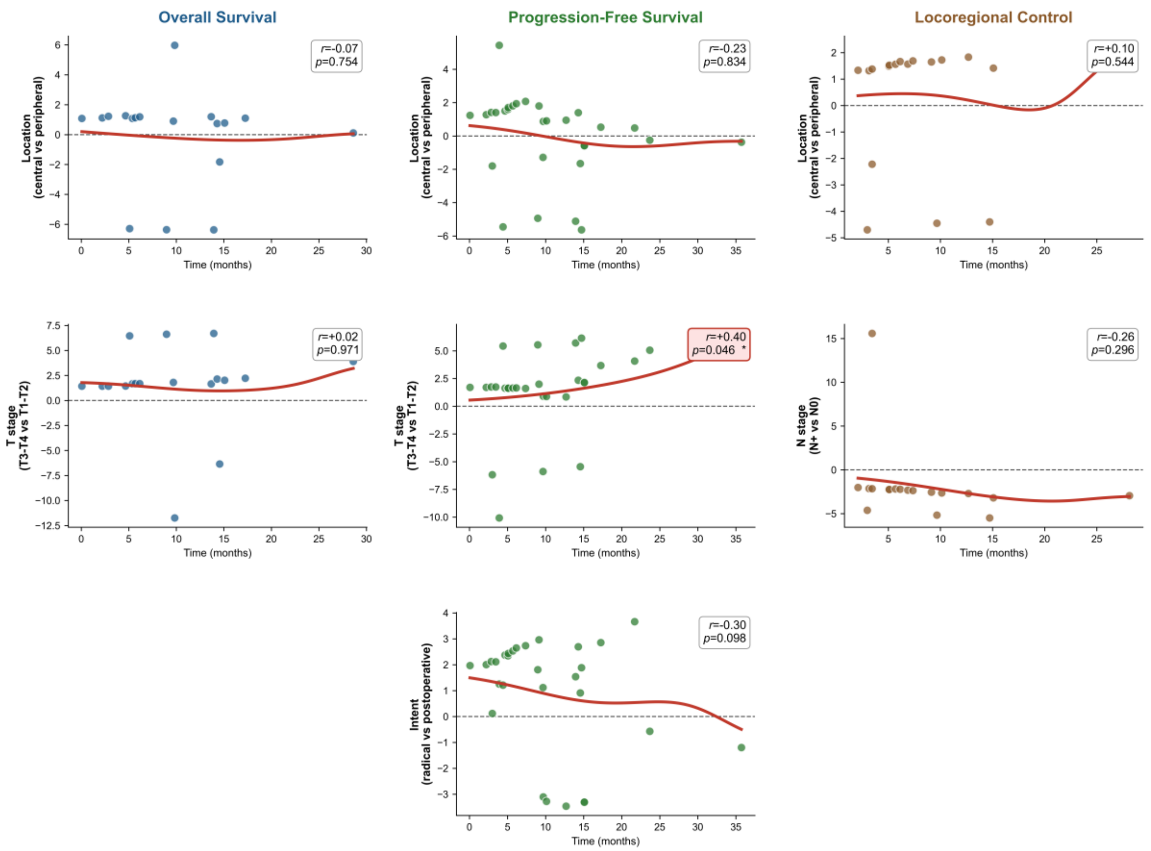

Figure S1. Scaled Schoenfeld residuals for the multivariable Cox proportional hazards models: Scaled Schoenfeld residuals are plotted against event time for every covariate retained in the corresponding multivariable Cox model (OS: location, T-stage; PFS: location, T-stage, treatment intent; LRC: location, N stage). Red lines: locally weighted (Gaussian-kernel) smoother. A non-significant correlation ($p > 0.05$) supports the proportional hazards assumption; the only violation was T-stage in the PFS model (p = 0.046, highlighted), consistent with the exploratory nature of the multivariable analyses.

Table S1. Patients included, events and censored observations for each time-to-event endpoint ($n = 61$).

Endpoint	Patients included	Events	Censored
Overall survival	61	25	36
Progression-free survival	61	39	22
Locoregional control	61	22	39

Four of 65 patients were excluded owing to the absence of any recorded follow-up or death date. PFS events comprise 26 documented disease progressions and 13 deaths without prior progression. For LRC, death without locoregional failure was treated as a censoring event.

Table S2. Baseline characteristics by anatomical extent of re-irradiation (n = 65).

Variable	Central/skull base (n = 45)	Peripheral (n = 20)	p
Median age, years (range)	60.2 (18–80)	62.2 (18–89)	0.565
Male sex	32 (71.1)	11 (55.0)	0.260
Squamous cell carcinoma	29 (64.4)	18 (90.0)	**0.039**
T3–T4 stage [i]	31/35 (88.6)	5/12 (41.7)	**0.003**
Node-positive (N+)	8 (17.8)	10 (50.0)	**0.015**
Radical intent	33 (73.3)	12 (60.0)	0.383
Concurrent chemotherapy	12 (26.7)	6 (30.0)	0.773
Median prior RT courses (range)	1 (0–3)	1 (1–2)	0.144
Median prior cumulative dose, Gy (range)	66 (24–72)	66 (32–80)	0.970
Median interval prior RT → PT, months (range)	38 (11–300)	28 (14–262)	0.513

Data are n (%) unless otherwise stated. p-values from Mann–Whitney U test (continuous variables) or Fisher's exact test (categorical variables). Bold p-values indicate statistical significance ($p < 0.05$). [i] Among patients with documented T-stage (35 central, 12 peripheral). RT: radiotherapy; PT: proton therapy.
